# The Top Chinese Mobile Health Apps: A Systematic Investigation

**DOI:** 10.2196/jmir.5955

**Published:** 2016-08-29

**Authors:** Jeffrey Hsu, Di Liu, Ya Min Yu, Hui Tong Zhao, Zhi Rou Chen, Jiao Li, Wei Chen

**Affiliations:** ^1^ Division of Cardiology Peking Union Medical College Hospital Chinese Academy of Medical Sciences and Peking Union Medical College Beijing China; ^2^ Institute of Medical Information and Library Chinese Academy of Medical Sciences and Peking Union Medical College Beijing China

**Keywords:** mobile health applications, mHealth, medical informatics, China

## Abstract

**Background:**

China’s mHealth market is on track to become a global leader by industry size. The Chinese mobile app market and health care system have peculiarities that distinguish them from other app markets. To date, Chinese mHealth apps have not been systematically investigated.

**Objective:**

The objective of this study was to provide an overview of Chinese mHealth apps as of December 2015.

**Methods:**

We identified and investigated the most downloaded apps from the iOS and Android platforms. For each app, we analyzed and recorded its main service offered, mHealth initiative, disease and specialty focus, app cost, target user, Web app availability, and emphasis on information security. Standard descriptive statistics were used.

**Results:**

A total of 234 apps met the inclusion criteria and were investigated. The apps targeting nonhealth care professionals focused on providing telemedicine and appointment-making services. The apps targeting health care professionals focused on education and peer reviewed articles. The most common disease-specific apps focused primarily on diabetes, hypertension, and hepatitis management. Most apps were free and available on both iOS and Android platforms.

**Conclusions:**

The primary mHealth initiatives targeted by the apps reflect Chinese patients’ demand for access to medical care. Disease-specific apps are also representative of disease prevalence in China. Government press releases suggest that new policies on the horizon may shift the industry.

## Introduction

mHealth, an area of electronic health, is the provision of health services and information via mobile technologies such as mobile phones [1,2]. mHealth apps have epitomized the typical mHealth service. These apps have the potential to cut costs, promote patient engagement, and improve health outcomes [3]. Much has been reported about the services in developed countries [3,4]. To our knowledge, mHealth apps in Mainland China have not been systematically evaluated.

China’s mHealth industry is a rapidly growing sector with a year-on-year growth rate of 29% in 2014 and a forecasted growth rate of 49% in 2015 [5]. The market size is expected to reach ¥7.18 billion (approximately US $1.08 billion) in 2016 and ¥12.5 billion (approximately US $1.90 billion) in 2017 [6]. In China, patients often have difficulty gaining access to appropriate medical care [7-9]. mHealth has the potential to provide widely accessible services that can be individually tailored and easily adopted.

An understanding of the Chinese health care system and smartphone usage provides a framework for understanding mHealth apps in China. The Chinese health care system has peculiarities that make mHealth a viable option. On the surface, the overall Chinese medical system can be comparable with those in advanced countries. In 2011, the doctor to patient ratio was not profoundly different between China and other developed countries: 1.5 per 1000 patients in China versus 2.5 in the United States and 2.7 in the United Kingdom [10]. The imbalances within the Chinese health care system become apparent when comparing urban versus rural areas. Health care expenditure varied by nearly 4-fold in 2009 between urban and rural areas [11]. As the overall health care system grew between 1980 and 2006, the number of beds in rural areas actually decreased. Also, the quality of care was far inferior in rural areas: the infant mortality rate was 16.1% in rural areas versus 5.8% in urban areas [11]. Due to the imbalance of medical resources, patients flock to urban areas seeking medical resources. The displacement of rural patients to urban areas causes difficulties in obtaining access to high-quality care, since rural and urban patients all compete for access to the same medical resources.

The smartphone usage rate is also unique in China. In 2014, 62% of the Chinese population between 16 and 59 years old owned a smartphone. By city tiers, smartphone ownership among the same age range was 94% in tier 1 cities and 75% to 88% in tier 2 cities, while the rate in rural areas was 32% [12]. Also, the Android platform holds a strong foothold with approximately 70% share of the smartphone market [13]. The main Android app stores in China are operated by Baidu, 360, and Tencent; Google Play has been absent since a 2010 censorship dispute [14]. The app markets in China, whether iOS or Android, all have multistep quality and content screening before apps are showcased in the store [15-18]. However, the specific rejection criteria differ across each store and are summarized in Multimedia Appendix 1.

The purpose of this study was to provide an overview of the leading mHealth apps in Mainland China as of December 2015. This study investigated each app with regard to availability, service, and data security to understand the current state of the Chinese mHealth market. In this study, we focused on medical-related apps instead of general health care. Hereafter, mHealth refers to mobile apps as they pertain to medicine.

## Methods

### Selection of Apps

We sampled apps from both the Android (Google, Mountain View, CA, USA) and iOS (Apple Inc, Cupertino, CA, USA) mobile phone app stores. For Android, we sampled apps from the 3 largest Android app stores in China operated by Tencent (Tencent Holdings Limited, Shenzhen, China), Baidu (Baidu, Inc, Beijing, China), and 360 (Qihoo 360 Technology Co. Ltd, Beijing, China). The 3 stores make up nearly 60% of the Chinese Android market share [19]. For iOS, we used App Annie (App Annie, San Francisco, CA, USA) to gather the list on China’s iOS market. We obtained the sample of apps on December 5, 2015.

We collected the top 100 apps according to each app store. The Android stores listed free and paid apps together. The iOS apps separately listed free and paid apps; thus, we collected both the top 100 free and top 100 paid apps. We systematically evaluated the free apps and but did only a cursory assessment of the paid ones.

**Figure 1:**
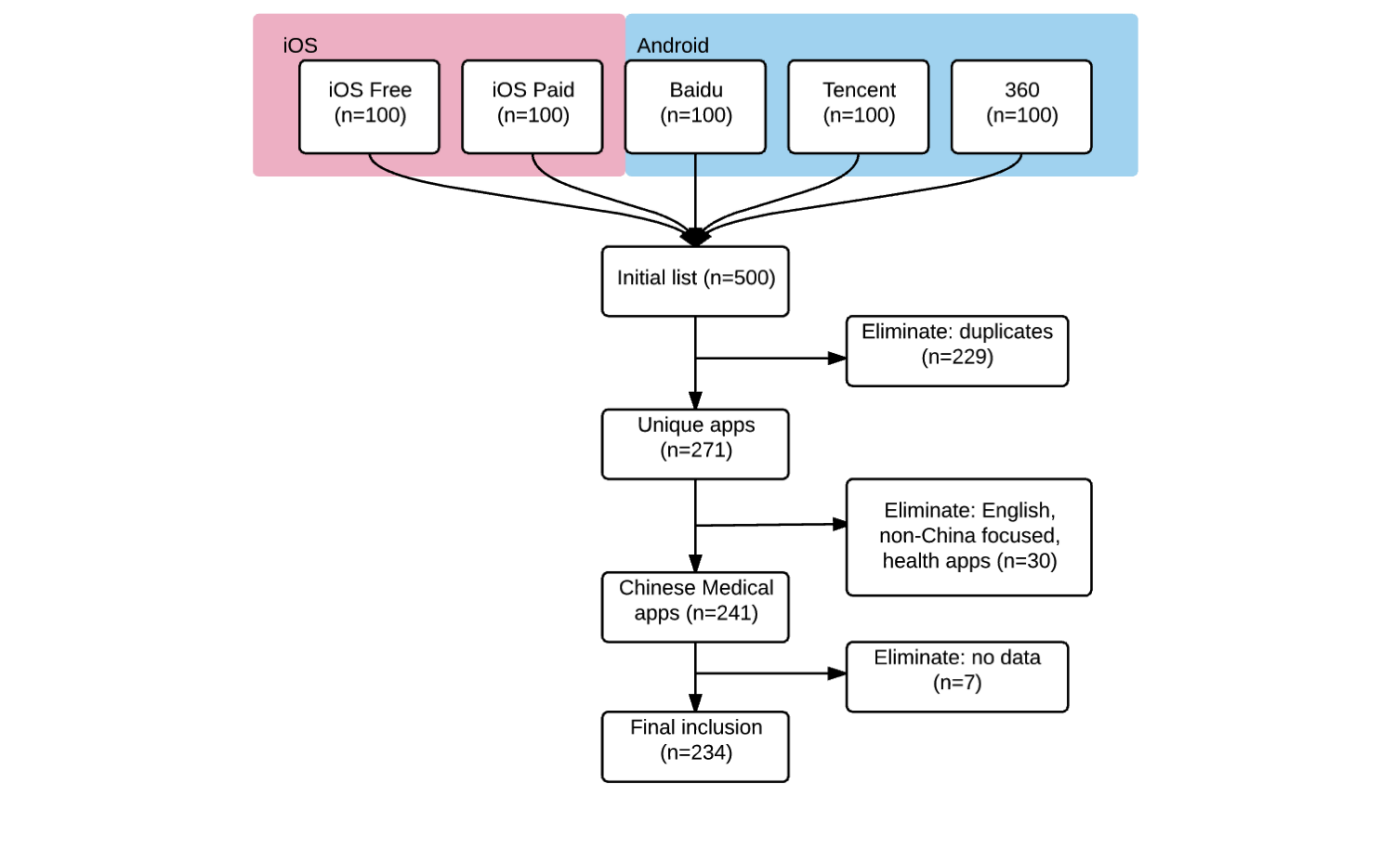
Flowchart of the selection process for Chinese mHealth apps.

We selected apps from the medical category for further evaluation. We then reviewed the apps for potential inclusion into the study. The inclusion criteria were as follows: simplified Chinese language, service tailored toward Mainland China (excluding Taiwan, Hong Kong, and Macau regions), and services pertaining to health care and medicine, not general health. For example, we omitted weight loss, exercise, smoking cessation, and menstrual cycle management apps (Figure 1).

The initial screening was completed by 4 authors: LD, YYM, ZHT, and CZR. The apps included for further evaluation were randomly assigned to these 4 authors. Each app was reviewed using information from the app store description, the app’s website (if available), and the app itself. We then reviewed each app collectively to ensure accuracy. If discrepancies existed, the particular app was discussed and a consensus was reached. For each app, we identified the main service offered, mHealth initiative, disease and specialty focus, app cost, target user, Web app availability, and emphasis on information security.

### Categorization of Apps

We recorded what service was offered based on the app store description and assigned the app to a corresponding mHealth initiative. mHealth initiative describes where an app’s service lies along the continuum of medical service delivery. In this study, we adopted the categorization of mHealth initiatives from 2 previous reports [1,6] and tailored it for the Chinese market. The 10 health initiatives used in this study were as follows: (1) appointment making, (2) reminders, (3) telemedicine, (4) records and patient monitoring, (5) pharmacy, (6) disease awareness, (7) clinical decision support, (8) discussion forums, (9) medical education and scholarly articles, and (10) other (Table 1). Each app could have one or more identified mHealth factors.

**Table 1:** mHealth initiatives previously reported and used in the categorization of Chinese mHealth apps in this study.

Previously published categories^a^	Categories adapted for this study
Call center	Appointment making
Reminders	Reminders
Telemedicine	Telemedicine
Records	Records and patient monitoring^b^
Treatment	Pharmacy
Awareness	Disease awareness
Patient monitoring^b^	Records and patient monitoring
Decision support	Clinical decision support
Discussion forum	Discussion forum
Scholarly articles	Medical education and scholarly articles
Other	Other

^a^Sources: Ryu [1] and Xiaohui et al [6].

^b^In previous reports, patient monitoring was an individual factor. In this study, patient monitoring was categorized together with records.

We assessed the disease and medical specialty of an app by the app’s name, app store description, and website. We matched the diseases we identified to its closest *International Classification of Diseases, Tenth Revision* counterpart.

App cost referred only to the cost at download. Additional fees incurred after usage were difficult to systematically evaluate and thus were not considered. We considered cost to be a binomial variable.

For target users, we classified apps as being focused on health care professionals (HCPs), non-HCPs, or both. HCPs referred to those included in the World Health Organization’s health professional categorization [20]. Since clinicians in public Chinese hospitals are typically required to conduct research [4,21], we also included clinical research-focused apps, even though the WHO categorizes this profession under life sciences. Apps identified as “both” had an HCP and a non-HCP version available.

For Web app availability, we examined the app store and website to determine whether a Web-based version of the app was available.

Finally, we examined whether an app emphasized information security. China lacks an industry standard or legislation regarding medical information safety or privacy similar to the Health Insurance Portability and Accountability Act in the United States [22]. Thus, for information security, we evaluated whether each app self-reported relevant information security measures. We labeled information security for each app as absent, present, or complete. For apps to have complete information security, they had to present documentation or a link referencing a third-party auditor.

Categorizations were made based on the description in the corresponding app store. For apps that were present in multiple app stores, we ensured the consistency of the descriptions across stores and eliminated duplicates. Some apps had a patient and a clinician version. We evaluated these as one entry. Descriptive statistics were used to describe the characteristics of the apps. Heat maps were used to identify the areas of the market receiving the most traction.

## Results

There were 241 unique apps that met the inclusion criteria. When we analyzed the apps, 7 were not available or had been taken offline, and thus we eliminated them from the final list. We analyzed a total of 234 apps. Of these, 195 were available in both iOS and Android app stores. However, 22 (9.4%) and 17 (7.3%) of the apps were available exclusively in the iOS and Android app stores, respectively.

**Figure 2:**
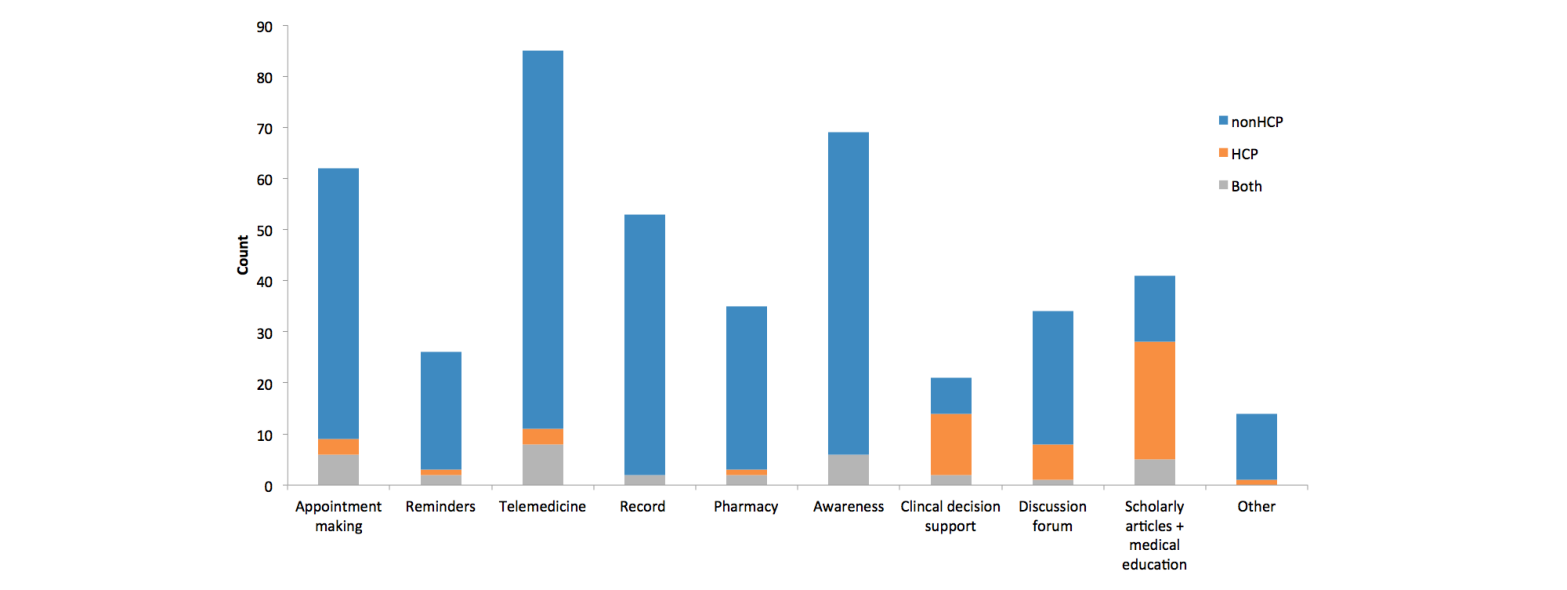
Distribution of Chinese mHealth apps according to medical initiative and user type. HCP: health care professional.

The most common medical initiatives were telemedicine, disease awareness, appointment making, and records and patient monitoring (Figure 2). The least common service factors were reminders and clinical decision support. Apps classified under other services included pharmaceutical drug information, drug delivery, insurance plans, and online-to-offline (O2O) health checkup services. We subdivided each medical initiative into the corresponding target user. Apart from clinical decision support and medical education, which primarily focused on HCPs, all other health initiatives were mainly aimed at non-HCP users.

**Figure 3:**
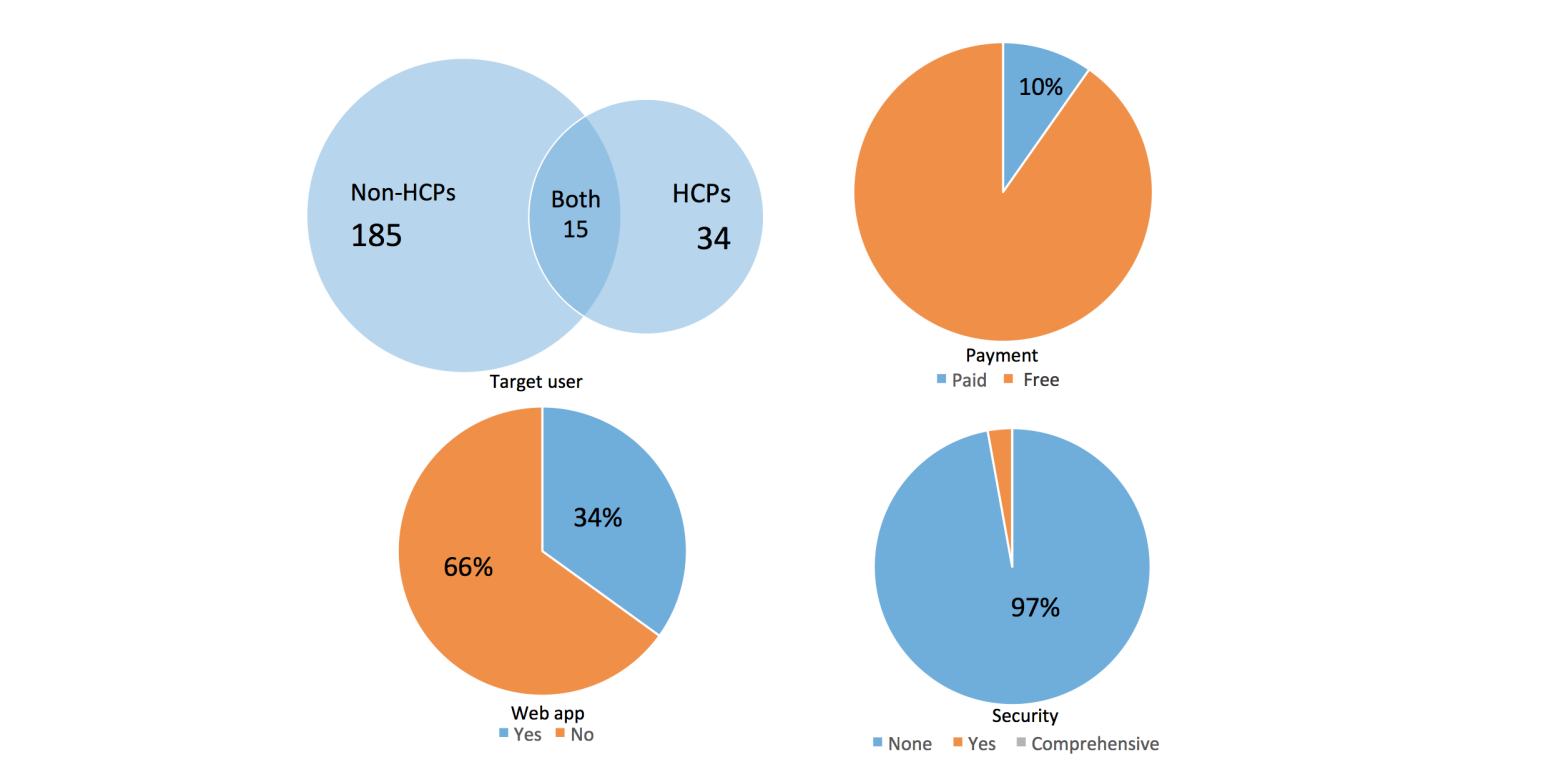
Features of the Chinese mHealth app landscape. Top left: Venn diagram of Chinese mHealth apps’ target users; top right: distribution of paid and free apps; bottom left: portion of apps with and without a Web-based version; bottom right: portion of apps with medical information security measures. HCP: health care professional.

Of the apps, 185 targeted non-HCPs, while 34 targeted HCPs, and 15 had both versions available. A total of 210 (89.7%) of the mHealth apps in China were free. All the paid apps were from the iOS app store. Nearly one-third (154/234, 65.8%) of the apps had both a mobile and a Web-based version. 227 of 234 apps (97.0%) of the apps did not mention information security (Figure 3). Of the 7 apps (3.0%) that mentioned information security, none had undergone external auditing.

**Figure 4:**
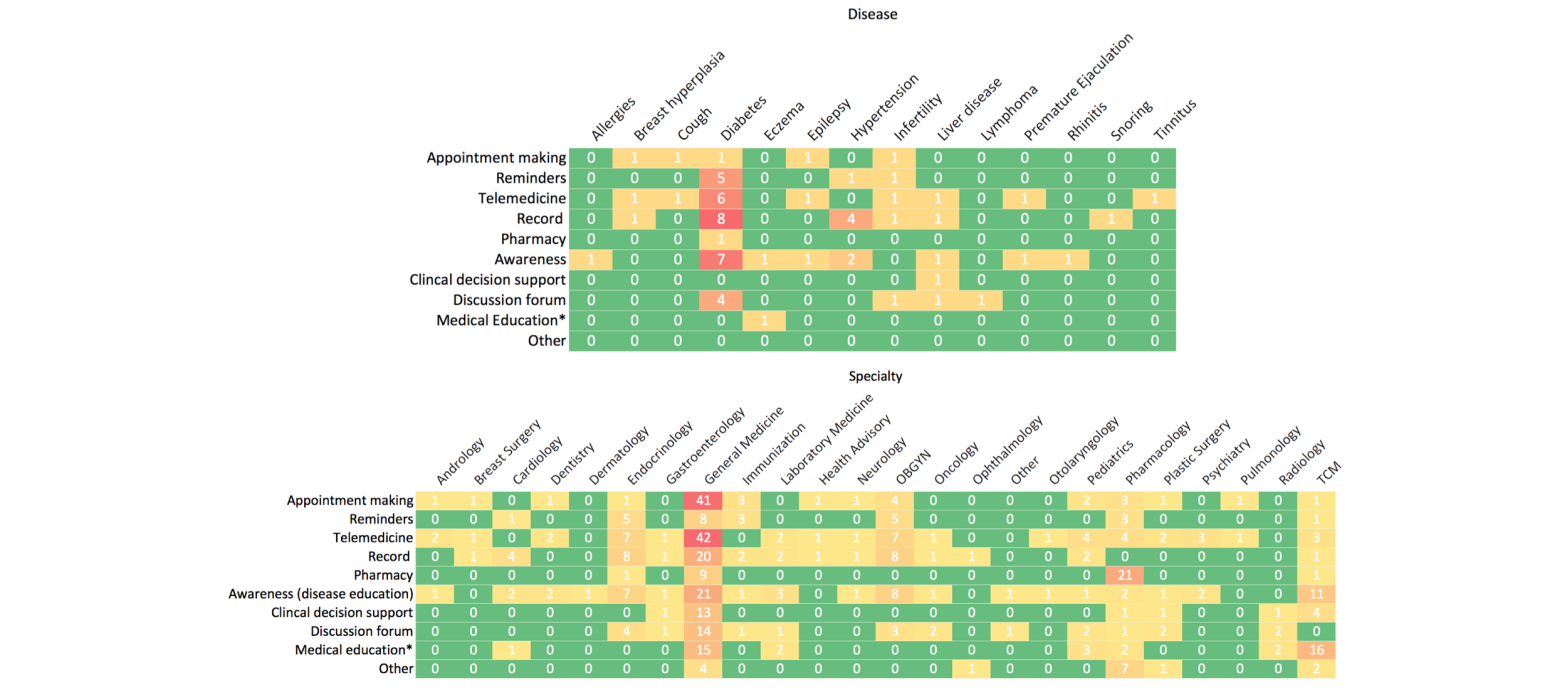
Heat map of medical initiative targeted by applications from (top) each disease and (bottom) medical specialty. Note white numbers refer to the count of apps within each specialty. Warmer colors refer to higher count. *Medical education includes dissemination of scholarly articles. OBGYN: obstetrics and gynecology; TCM: traditional Chinese medicine.

We created heat maps to examine the distribution of apps along the medical initiative, and the disease and medical specialty spectrum. The most common diseases were diabetes, hypertension, liver disease (general), and infertility (Figure 4, top). For diabetes, the apps were focused on record keeping and patient education. The most common specialties were general medicine, obstetrics and gynecology, endocrinology, pharmacology, and traditional Chinese medicine (Figure 4, bottom). Apps classified under general medicine covered an assortment of specialties without an emphasis on any particular one.

## Discussion

### Principal Findings

This study provided a snapshot of the Chinese mHealth industry in December 2015. The primary mHealth initiatives targeted by the apps reflected Chinese patients’ demand for access to medical care. The primary initiatives were telemedicine, disease awareness, appointment making, and record keeping, followed by medical education and scholarly information. The overwhelming majority of the apps were not specific to a medical specialty. However, the apps that targeted a disease reflected prevalent conditions in China such as diabetes, hypertension, and hepatitis. The target users among apps were mainly non-HCPs. An overwhelming majority did not mention information security. We discuss each aspect further below.

#### mHealth Initiatives

Through the heat map and mHealth initiative analyses (Figure 4), we found that among the most common mHealth initiatives were telemedicine, appointment making, and medical education. Telemedicine can broadly be defined as the use of telecommunications technologies to provide medical information and services [23]. The use of telemedicine to diagnose and prescribe medication has yet to be legalized in China. This implies that the actual service delivered in telemedicine was limited to general medical inquiries, with limited medical actions involved. Appointment making reflects a phenomenon across China of patients lacking access to clinicians [7,9].

Here, we discuss the role and background of hospital appointment making. In 2009, the Chinese Ministry of Health required all public hospitals of level 3 [24] or higher to allow appointment-making services and prohibited partnering with third-party agencies to profit from these services [25]. In recent years, for a given public hospital, available appointment dates have been provided directly on the hospital’s appointment-making system, public (municipal or provincial) third-party systems, or private third parties such as apps. (Of note, in the Peking Union Medical College Hospital in particular, the ratio of self-distributed to public third-party distribution is 7 to 3.) These apps obtain a number of appointments by signing agreements with hospitals or with individual doctors. Apps solve the problem of difficult access by (1) aggregating hospital appointment availabilities on their platform, thus preventing patients from waiting at fully booked hospitals or (2) partnering with individual doctors to provide consultations on personal free time.

Apps focused on medical education and scholarly articles targeted two fundamental demands in the Chinese health care market: access to reliable information, and clinicians’ need to publish peer reviewed articles. The proliferation of discussion forums and unregulated “medical” articles on the Internet and apps makes reliable information a rare commodity. The abundance of medical education apps appears to target this need.

On the other hand, a clinician’s professional advancement is dependent on many factors, of which publishing peer reviewed articles is crucial [21,26]. The availability of apps to provide articles and assist with the writing process can be an attempt to supply this demand. These apps provide writing resources such as editing and actual writing, and provide access to scholarly articles. This access is often through a Chinese translation or a summary of the original article (most often in English).

#### Disease- and Specialty-Specific Apps

We examined the distribution of diseases and medical specialties in the mHealth industry through a heat map plotted against medical initiative. When examining the frequency of apps on a disease basis, we determined diabetes to be the most common, followed by hypertension and hepatitis. This is consistent with epidemiological surveys pointing to these diseases as the most prevalent in Mainland China. According to a report from the Chinese Center for Disease Control, the prevalence of hypertension among Chinese adults was 33.5% (amounting to roughly 330 million hypertension patients) [27]. A recent study showed the prevalence of type 2 diabetes at 11.6% of the population (amounting to about 90 million diabetes patients) [28]. Since mHealth apps for these diseases focused almost exclusively on disease monitoring and recording, market forces driven by pharmaceutical drug sales may not be the key factor for app providers. Rather, it may be the distribution of monitoring devices. Diabetes is monitored by glucometers and single-use disposable test strips, versus repeated-use blood pressure machines for hypertension. The market opportunity for diabetes in mHealth is believed to be greater than that for hypertension due to the nature of disease management and monitoring, despite hypertension having nearly triple the patient volume.

Unlike many developed countries in the world, in China hepatitis B is endemic. Approximately one-third of worldwide cases are in Mainland China [29]. A report noted 120 million carriers of hepatitis B virus in China and 30 million patients who are chronically infected [30]. The existence of apps targeting hepatitis is consistent with the disease’s endemic nature in China.

The heat map of medical specialty by health initiative provided more information about the industry as a whole. By far the most heavily targeted specialty, or lack thereof, is general medicine. The apps here mainly focused on appointment making and telemedicine. The telemedicine services offered were often a “lite” version of medical history taking, since patients were provided with general answers and then encouraged to seek specific guidance through in-app appointments. Thus, despite telemedicine and appointment making having lexical and implicit differences, Chinese mHealth apps providing telemedicine services were more synonymous than they appeared based on the above analysis. This finding reinforces the difficulty of accessing care.

Another popular area was pharmacology. The apps in pharmacology provided patients with access to online or offline pharmacies and provided clinicians with pocket drug references. Pharmacology-related apps aimed to provide convenience for patients who require drug refills and cannot repeatedly travel to community pharmacies or hospitals. Community pharmacies often do not carry a full repertoire of prescription drugs, and the current state insurance policy covers in-hospital prescriptions for 14 days for able-bodied patients and up to 30 days for disabled patients. On the other hand, pharmacology apps targeting clinicians, pharmacists, and other HCPs provided references for dosing, interactions, and alternative drugs. The incorporation of pharmacology apps into HCPs’ daily practice appears to be a mainstay in China and many other countries [31,32].

#### Web App

Accessing an app from different media can allow users to experience the app differently and can serve different purposes. The apps in this study focused on mobile phones. However, about one-third of the apps also had a Web-based version. The main difference between the Web app and mobile app was the ability for users to view historical data organized in reports or graphs in the Web-based version. It has been reported that data visualization and context awareness could enhance an app’s utility [33].

#### Target User and Payment

An app’s target user can provide information about market opportunities and underlying market forces facing app developers. Our analysis showed that app developers preferred targeting the non-HCP user, perhaps due to easier user acquisition and a larger potential user base. The HCP-oriented apps mainly provided services through medical education or scholarly articles. Few focused on medical care delivery or integration into hospital care. This can be explained by financial compensation related to publication requirements [21,26]. A physician’s compensation is directly tied to his or her position in a hospital hierarchy. Among many factors, publication quotas are mandatory evaluation metrics in many hospitals. We believe that this demand on physicians compliments an app developers’ need to grow and retain a steady user base. There were 10 apps with both an HCP and a non-HCP version available. These apps all allowed communication between the two parties.

Most apps in China were free for users to download and use. All apps surveyed from the Android app stores were free. Chinese users are likely accustomed to using free apps.

#### Security

We examined the lack of information security from two perspectives: market forces and government regulations. Nearly all apps failed to mention on their website or user agreement form about securing the users’ information. This can imply gross negligence from the developer or use of the data for other purposes. The latter is likely the underlying motive because, as noted by notable industry agencies, information can be knowingly or unknowingly sold to marketers for financial gains [34].

We also considered information security from a policy perspective. We examined policies pertaining to medical apps by reviewing documents from the People’s Republic of China State Council’s official website [35] dated between 2013 and 2015. The search was limited to “Internet,” “Internet plus health care,” and “Internet plus medicine.” All results were read and screened by a Chinese Health Care Policy analyst (author LD) for relevance. Among the more than three thousand policies and documents available, no direct laws governing medical apps, let alone mHealth security, were found, and only 11 official releases were identified that can directly or indirectly affect the operation of medical apps (Table 2).

**Table 2:** Documents^a^ by the Chinese State Council or General office of the State Council related to the governance of mHealth.

Document number	Document content	Targeted industry
1	Internet plus action plan	Manufacturing, transportation, information technology
2	Action outline on promoting the development of big data	Manufacturing, transportation, information technology
3	Medical services and health care system planning guideline (2015–2020)	Health
4	Circular on boosting the development of e-commerce	Comprehensive government affairs
5	Guideline on strengthening support for consumer services to upgrade consumption	Commerce
6	Guideline on further boosting consumption as a key component in driving economic development	Commerce
7	Guideline on pushing integrated medical and nursing care for the elderly	Health
8	Circular on strengthening the Patriotic Hygiene Campaign in the new era	Health
9	Opinions on using big data technology to improve the government’s supervisory responsibilities and services for market entities.	Manufacturing, transportation, information technology
10	Opinions on cracking down on infringement of intellectual property rights and the production of fake and inferior commodities in cyberspace to safeguard the healthy development of e-commerce	Science, education, intellectual property
11	The legislation working plan of 2015	General

^a^Source: State Council of the People’s Republic of China [35].

The scope of mHealth apps can cross into many industries. Documents mentioning mHealth targeted industries such as information technology, manufacturing, health care, commerce, and intellectual property. The fact that many policies, across various industries, mention mHealth suggests that the Chinese government recognizes the future potential of the industry but has yet to dictate a clear stance on how to regulate this relatively new industry. The lack of specific policies toward mHealth may be due to the difficulty in pinpointing which industry it lies in.

#### Platform

Most apps were available on both app platforms. Android dominates the Chinese smartphone market at over 70% market share [13]. The iOS app store is known to have more apps available than the Android store. However, many are not specific to the Chinese market. It is possible that mHealth app companies are developing the apps domestically and targeting consumers on both platforms.

### Limitations

There are limitations to this study. First, the number of apps sampled is small and cannot fully explain the market. This study was meant to provide a snapshot of the industry as a whole rather than details along each medical specialty. To fully understand the availability and characteristics of the apps for each specialty, the analysis should be done in disease or specialty verticals. Second, this study focused on medical apps while excluding general health apps. This allowed for a more homogeneous analysis, since apps targeting healthy users and sick users are likely different in nature. However, we excluded a large portion of apps from the analysis.

### Prospective

In the future, a shift in mHealth apps from delivering purely online services to an O2O approach is expected. O2O, a concept common in Chinese e-commerce, refers to an integration of offline businesses into online commerce [36,37]. Online services in health care can refer to mobile apps, websites, or other digital tools. Offline services include services delivered in physical sites such as hospitals, clinics, pharmacies, and health centers. The idea of O2O in mHealth refers to the integration of online services delivered via apps with “traditional” health service providers. Possible applications include prescription apps that allow patients with existing prescriptions to have their drugs delivered by a local pharmacy, and third-party apps that provide electronic health records services linked to the electronic medical records of a regional clinic.

As of July 2016, the government released legislation banning mHealth apps from providing appointments for patients. The legislation requires appointments to be made directly through the hospital, not a third-party provider. The reasons behind the ban were to ensure the integrity of the hospitals and protect patients’ interests. Most Chinese hospitals are nonprofit public institutions. Providing public resources to partnering companies for financial gain violates their nonprofit nature. In addition, third-party appointment-making apps commonly tack on a cost premium for each service-seeking patient. Finally, allowing third-party appointment-making apps to serve individuals who can afford a premium is a detriment to nonpremium-paying patients. Thus, it behooves the government to ban this service to protect the general public and prevent private-public partnerships from hoarding publicly available resources.

A growing trend, internationally and domestically, is allowing clinicians to send prescriptions via apps. Services offered in this space have obvious financial incentives and safety risks. The development of the prescription app industry is expected to catch the government’s attention. There is precedence for the government to interject and control specific health markets. How or when that will occur is to be determined. As policy shifts loom on the horizon, mHealth providers must react. One common theme found in China and in the United States is that the “grassroots entrepreneurial nature of the market” [3] appears to be the main driver of the mHealth industry.

### Conclusion

mHealth in China is a large and continuously growing market. The potential to disrupt the traditional health care market exists. At the end of 2015, the Chinese mHealth market targeted the nonprofessional user. The services offered heavily focused on the demands of HCPs and non-HCPs, such as publishing peer reviewed papers and gaining access to clinicians, respectively. Also, a unifying policy or standard from the Chinese central government or the China Food and Drug Administration to govern this industry is lacking, but evidence shows that the government is cognizant of the potential this industry and regulations may have in the near future.

## Appendix

Multimedia Appendix 1 Common reasons for app rejection across iOS and Chinese Android app stores. Android app stores include 360, Baidu, and Tencent.

## Abbreviations

HCP: health care professional
O2O: online-to-offline
