# Supplementary figures and images for: The Top Chinese Mobile Health Apps: A Systematic Investigation
Source: J Med Internet Res. 2016 Aug 29;18(8):e222. doi: 10.2196/jmir.5955 (PMC5020314; doi:10.2196/jmir.5955)

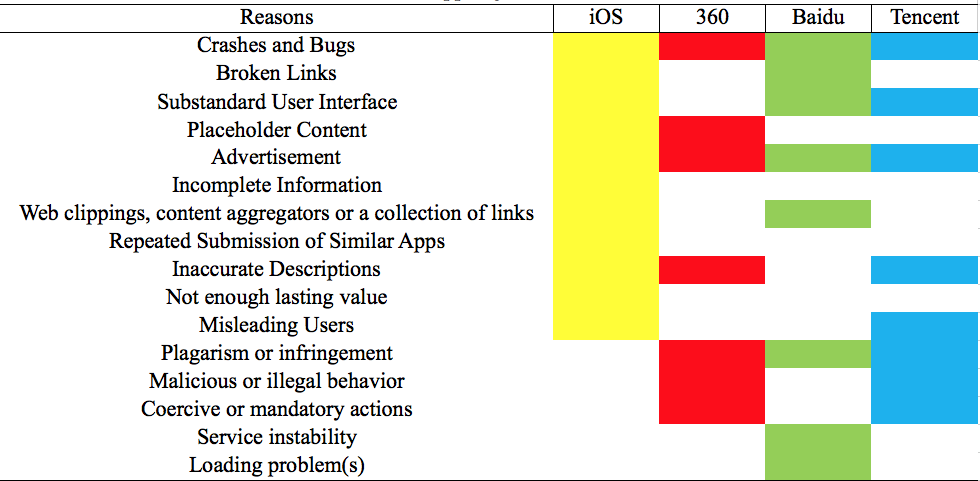

Supplement: Multimedia Appendix 1 [file jmir_v18i8e222_app1.png]
